# Tabula rasa agents display emergent in-group behavior

**DOI:** 10.1073/pnas.2319947121

**Published:** 2025-06-16

**Authors:** Raphael Köster, Edgar A. Duéñez-Guzmán, William A. Cunningham, Joel Z. Leibo

**Affiliations:** ^a^Google DeepMind, London EC4A 3TW, United Kingdom; ^b^Department of Psychology, University of Toronto, Toronto, ON M5S 3G3, Canada; ^c^Vector Institute, Toronto, ON M5G 1M1, Canada; ^d^Schwartz Reisman Institute for Technology and Society, University of Toronto, Toronto, ON M5G 1L7, Canada

**Keywords:** AI, multiagent reinforcement learning, group bias, cognitive models, social psychology

## Abstract

Theories on group-bias often posit an internal preparedness to bias one’s cognition to favor the in-group (often envisioned as a product of evolution). In contrast, other theories suggest that group-biases can emerge from nonspecialized cognitive processes. These perspectives have historically been difficult to disambiguate given that observed behavior can often be attributed to innate processes, even when groups are experimentally assigned. Here, we use modern techniques from the field of AI that allow us to ask what group biases can be expected from a learning agent that is a pure blank slate without any intrinsic social biases, and whose lifetime of experiences can be tightly controlled. This is possible because deep reinforcement-learning agents learn to convert raw sensory input (i.e. pixels) to reward-driven action, a unique feature among cognitive models. We find that blank slate agents do develop group biases based on arbitrary group differences (i.e. color). We show that the bias develops as a result of familiarity of experience and depends on the visual patterns becoming associated with reward through interaction. The bias artificial agents display is not a static reflection of the bias in their stream of experiences. In this minimal environment, the bias can be overcome given enough positive experiences, although unlearning the bias takes longer than acquiring it. Further, we show how this style of tabula rasa group behavior model can be used to test fine-grained predictions of psychological theories.

## Significance

The emergence of group bias has a long history in social psychology, including the positing of innate biases. Experimental evidence is fundamentally limited because every brain is both a product of experience and evolution. With cognitive models, one can control both the cognitive architecture and experience that shape its learning. We show that multiagent reinforcement learning can vastly expand the social scenarios that can be studied using a model that learns “from scratch.” This is possible because deep reinforcement learning agents model reward-guided decision-making and learn to parse the world from raw sensory input. Here, we explore the emergence of group bias in simulation and the factors mediating it. Importantly, these biases are overcome with sufficient experience.

People form social groups to facilitate coordination and protect themselves from various threats, including environmental challenges and other people (1, 2). Trust within these groups facilitates long-term cooperation while group boundaries facilitate coordination with unfamiliar individuals given shared fate (3). While the formation and maintenance of social groups can provide clear advantages, the categorization of individuals into in-groups and out-groups can lead to negative consequences, particularly when group delineations are arbitrary or associated with overgeneralizations, false beliefs, and differences in power and resources. This raises the important question of how and why people form specific groups, especially when the boundaries appear arbitrary, do not serve any immediate interests, and when people report egalitarian motivations to avoid these types of social bias (4). Social bias and in-group favoritism are often explained by one of four mechanisms: evolutionary modules that precondition people to seek in-groups and avoid out-groups (5, 6), social learning of personal identities (7, 8), power/dominance structures (9, 10), or as the accidental byproduct of general-purpose learning systems (11). These explanations are not mutually exclusive, but given people’s rich experiences, it is often difficult to disentangle the contributions of each mechanism and their contribution to the bias. Indeed, even with studies that use artificial groups and learning tasks, participants arrive with a wealth of experience with groups from which they can generalize social cognitive heuristics. To alleviate these issues in previous research, we propose a solution with simulated artificial “tabula rasa agents” to isolate mechanisms of social bias. These agents implement generic learning algorithms that learn to maximize their own reward in a given environment.

## Intrinsic Motivational Systems.

Although group bias can emerge from histories of conflict and social learning, research over the last century has documented the ease with which people develop and maintain group categories and in-group favoritism. In 1954, Gordon Allport noted that people who hold prejudices toward social out-groups also reported dislike for Wallonians, a fictitious group invented for the study only appearing on the questionnaire (12, 13). Even more dramatically, studies using the classic Minimal Group Paradigm have demonstrated that people readily form in-groups and out-groups based on arbitrary and meaningless criteria, such as preferences for abstract paintings (14). Simply assigning people to groups, even when the arbitrary nature of the assignment is made known to the participants results in differences in how much money is allocated to members of their group vs. other groups (14–16). Recent work has shown that this assignment difference can manifest in automatic evaluative responses (17) and changes to the neural responses to viewing faces of arbitrarily assigned in- and out-group members (18). Interestingly, these effects appear to be driven primarily by associating in-groups with positivity rather than negativity to out-groups (19, 20). To account for effects like these, researchers have proposed various motivational forces that drive intergroup cognition. For example, Tooby and Cosmides’ coalitional instincts hypothesis suggest that the near-universal way that people sort themselves into in-groups and out-groups arises from evolved specialized cognitive mechanisms that detect and respond to social alliances which provided significant evolutionary advantages to our ancestors in terms of survival and reproduction (21, 22). Similarly, out-group bias has been explained through evolved mechanisms designed to detect and avoid potential sources of infection (5).

Other theoretical accounts share the assumption that innate motivational systems are involved in intergroup cognition without requiring specialized group-attentive mechanisms. Most prominently, Tajfel and Turner (23) proposed that people have a natural desire to maintain a positive self-image and boost their self-esteem. On this view, as social groups become associated with one’s identity, people will enhance the status of their own groups and denigrate others as a means toward enhancing their self-image (24). Research has demonstrated greater in-group bias when one’s identity within the group is weak or threatened (25). Interestingly, these social bias-identity effects have been proposed to not require conscious awareness and may arise simply by fusing one’s self-esteem and group membership in an associative network (4, 26).

## Generic Cognitive Mechanisms.

The evolutionary origin of group dynamics and particularly altruism has been subject to a large amount of scholarship spanning different fields (27–29). Here, we want to model an independent or additional account of these group-level phenomena, arising purely from generic cognitive mechanisms. Although social group biases may have motivational origins, work in social cognition has highlighted that social biases can emerge as byproducts of general cognitive mechanisms and architectures of mind, such as learning, perception, and categorization. This line of work highlights how standard cognitive mechanisms such as categorization, generalization, and familiarity play out in social environments (11, 12, 30, 31). Providing experimental evidence for this idea, Hamilton and his colleagues conducted a series of studies that demonstrated how people tend to perceive an illusory correlation between group membership and negative behaviors. In these studies, participants were presented with information about members of two groups (majority and minority) engaging in positive and negative behaviors. Even when the ratio of positive to negative behaviors was equal for both groups, participants still perceived a stronger association between the minority group and negative behaviors (32). Mechanistically, features of majority group members have been shown to be learned more quickly than those of minority group members, and because of this features that distinguish majority from minority group members receive greater attention in learning resulting in more stereotypic associations for minority group members (33). To the extent that people often have far greater experience with their own groups, such mechanisms are likely to contribute to in-group biases.

People tend to prefer people, places, and things that they have had more contact with than ones that they have not, all other things being equal. For example, when participants are presented with novel stimuli (nonsense words or unfamiliar symbols) for a different number of exposures, participants self-reported greater liking for the stimuli that were presented more often (34). Indeed, this effect, known as the Mere Exposure Effect, has been replicated for subliminally presented stimuli suggesting that people do not need to be consciously aware of differences in exposure to develop these preferences (35). To the extent that people are generally more exposed to members of their in-group, as they share more experiences, social circles, and cultural contexts with them, it follows that this increased exposure leads to greater familiarity, which in turn fosters positive feelings and preferences toward in-group members. Conversely, individuals typically have less exposure to out-group members, which results in less familiarity and possibly more uncertainty or discomfort. To the extent that initial preferences can then determine who or what people chose to interact with for subsequent interactions (10), even small differences in initial preferences can cascade into larger biases. Once bias has emerged, people are often reluctant to interact with people from other groups (10, 36). The likely causal role of exposure for intergroup bias is further highlighted by research that has demonstrated that when people have the opportunity for positive and rewarding contact (37), initial biases decrease and trust and cooperation increase (38–40).

This focus on experience (i.e. exposure) aligns closely with the cognitive model of reinforcement learning, in which actions are incrementally shaped by repeated rewarding experiences. Here, the amount of experience with one group or another is important because it leads to more quickly learning the accurate reward value (e.g. if interacting with other people is on average of positive reward). Further, additional interactions may reduce uncertainty in the reward-estimates, even if experiences with both groups are equally rewarding. Indeed, neuroscientific research has shown that the mere exposure effect is tied to dopaminergic circuitry (41). Additionally, multiple lines of work point toward a dopaminergic role in stabilizing repeated actions into habits (42–44).

## Current Study.

The key challenge in attempting to decompose the mechanisms that give rise to intergroup behavior is that multiple theories can account for the same data. Empirically studying humans involved participants who typically had years of experience in the world driven by social learning. Having learned about group-dynamics in the real world could create heuristics that cause experimental observations like minimal group effects (rather than innate heuristics causing real world group-bias).

A simulation with cognitive agents allows to both control the stream of experience each agent receives, and the initial cognitive architecture each agent is furnished with. As we will detail below, recent advances in AI allow us to broaden the toolkit available for simulations of social behavior. In this model, motivational factors can be explicitly defined by the experimenter. By using agents that are merely equipped with a generic learning architecture that captures the cognitive process from perception of raw stimuli (i.e. raw pixels) to reward motivated action, we can examine whether group bias can be driven simply by differences in familiarity between groups. Further, to the extent that group-bias arises from simple perceptual and reward processes, we can examine factors that lead to greater or lesser bias, and how additional experience may be able to overcome bias.

## Multiagent Deep Reinforcement-Learning.

Many cognitive models offer “unbiased” learning, but mostly operate only in very constrained settings. For example, pure RL models used in psychology typically have the action and state space predefined by the researcher (and must therefore be relatively small). Deep reinforcement learning models vastly expand the capabilities of the types of tasks the cognitive model can participate in. A deep RL agent can work from raw visual input to base level actions (e.g. up, down, left, right) and merely needs rewards from the environment to optimize its behavioral policy. The behavioral policy of the agent is stored in a neural network, that layer by layer transforms the raw visual input into an action. Initially, this neural network has random weights. Over time, using the reward signal, learning gradually reshapes the neural network to parse the world into abstractions that are relevant for the task, to support reward-guided actions. This generality of the learning agent allows researchers to move from small, well-defined tabular games to complicated settings more akin to video-games. This provides a large benefit to the scope and complexity of behavior that can be modeled. In fact, deep reinforcement learning algorithms can even operate in situations where the researchers themselves do not know in advance how to program an effective behavior for the environment in question. Previous studies using these methods have explored economies (45, 46), the management of common-pool resources (47), provision of public goods (48), and the spread of social norms (49, 50). However, deep RL agents do not just offer a wider range of tasks they can process, but they also hold promise as a cognitive model of representation learning in the context of reward-guided action. Although not a complete model of human neural functioning, convolutional neural networks used mostly for image recognition have been used as a model for the conceptual hierarchy of processing in the visual stream (51), while RL models have been widely used to model the important decision-making processes in the brain (52). Combining them into one model allows to investigate how representations of a visual world are formed to support reward-guided decision-making (53–55). These models capture the important theoretical computational principles that are proposed to give rise to many psychological outcomes despite not being fully biologically plausible. Although psychological experiments have demonstrated the ways that people can form new attitudes toward social groups in laboratory settings, every participant (whether humans or nonhuman animals) brings a brain and a set of heuristics to generalize from to the experiment that has been shaped by a lifetime of experience. Deep RL allows us to conduct psychology experiments on these artificial agents. We suggest that these neural network architectures can serve as a simplified model for learning processes in the brain that start “from scratch.” By deploying multiple agents in the same complex environment, we get a “null-model” for social interaction. Such simulations isolate the interaction during development (for the artificial brains) as the only possible mechanism for social biases in their final behavior since we know they were not endowed with specific social biases. We know the individuals begin their lives “tabula rasa,” so any biases that emerge must be due to their experience.

Here, we set out to study what group in-group behavior would develop from tabula rasa agents. In particular, we want to investigate the effect of groups of agents that can be visually differentiated and who have different amounts of experience with their “in-group” vs. a potential “out-group,” and test whether this difference in experience leads to in-group behavior.

**Fig. 1.**
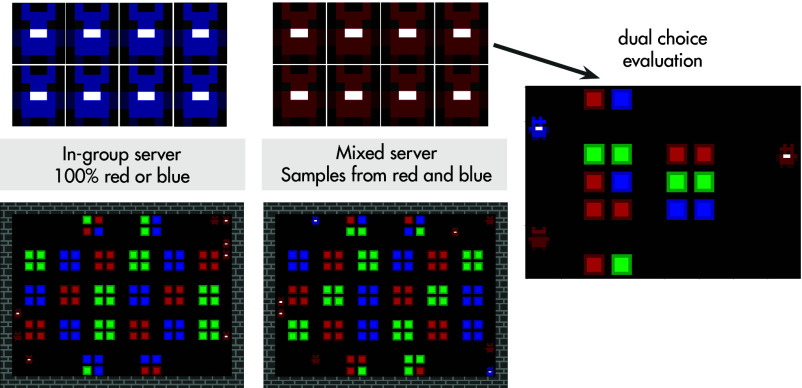
Schematic overview of the experimental design. The red and blue population interact in two “servers” that run the same underlying coordination task. One game server samples all the red agents or all the blue agents homogeneously; the second server samples from both populations (the exact ratio is varied across conditions). The dual choice evaluation measures the group bias by placing each agent (on the *Right* side of the environment), during training, in a separate environment and chooses whether to interact with an immobile sprite of the red or blue agent (on the *Left*). There is no reward-based reason to show a bias toward interacting with one color more than another.

We consider a population of 8 red and 8 blue agents (Fig. 1). They interact in two “servers” that run the same underlying task. The underlying task is a coordination game in which agents collect colorful resources and then can choose to “interact” with each other. If they do, they get reward in proportion to how similar their inventory of collected resources is. The “in-group” server samples all the red agents or all the blue agents homogeneously. On this server, agents increase their familiarity with agents from their own group. The “mixed server” samples from both populations, at a ratio we vary across conditions (8:0 to 4:4). To evaluate each agent, we take a “frozen” copy of the agent at different training steps (i.e., this copy is not learning during evaluation). We then place this frozen agent in a separate environment in a separate environment in which they are faced with a dual choice whether to interact with a red or blue agent. Importantly, as the color of agents is entirely unrelated to game dynamics, there is no “rational” reason to distinguish agent by color. Any observed group-bias, would be artifact of incremental, multiagent learning dynamics.

We show that tabula rasa agents display a group bias that is mediated by their experience with the different groups. This effect is mediated the amount of experience with the other group, the visual similarity between groups and whether rewarding interaction are possible. We also show that group bias is increased when agents have difficulty individuating a less desirable member of the out-group. Critically however, this group-bias in tabula rasa agents can be overcome with sufficient experience.

## Results

**Fig. 2.**
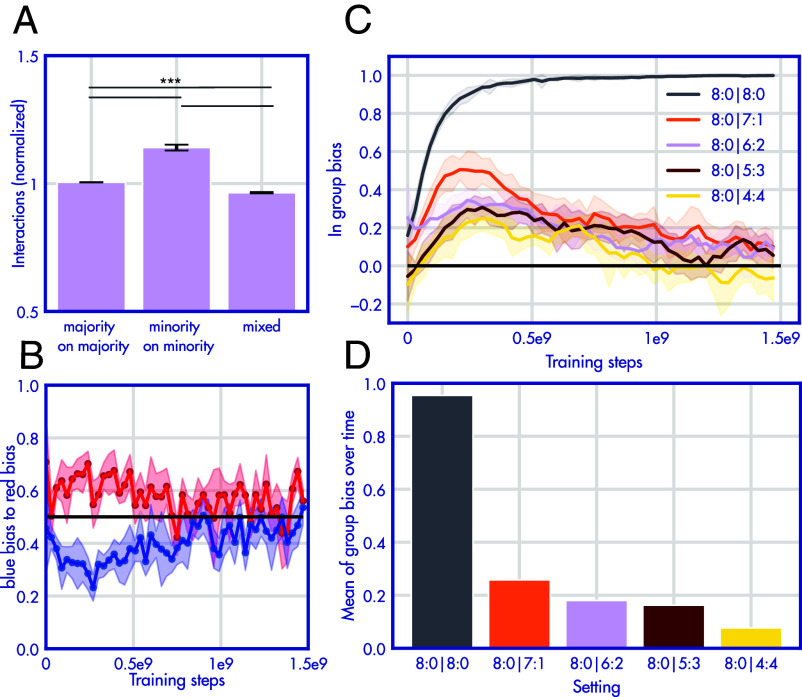
A group bias emerges from familiarity. (*A*) During training, agents interact more with agents that share their color (the plots are generated from 5 runs where 6 agents interact with 2 agents of the other color on the mixed server). The y axis shows interactions with agents per color (normalizes over frequencies expected from random interactions). For (*B* and *C*), the x axis is learning steps. (*B*) Shows data from the dual choice evaluation. Red agents show a bias of interacting with red agents, while blue agents show a bias of interacting with blue agents. This difference decays over time. (*C*) Shows the group bias (the gap between the blue and red line in *B*) for 5 different settings in which the mount of experience with the other group is varied. If 100 % of interactions are with their own group, agents show a maximal group bias. If agents have any exposure to the other group, their bias declines over time. (*D*) Shows the sum of bias in (*C*). There is a strong relationship between the amount of experience agents have with the other group and the group bias they display. All error bars show 95% CIs.

We first consider agent behavior in the training environment, where they need to coordinate with others to gain reward in an interaction. To investigate the patterns of interaction, we collapse the subgroups: For example, “majority on majority” combined red agents interacting with red agents (when there are 6, i.e., a majority of red agents in an episode) and blue agents interacting with blue agents (when there are a majority of blue agents). We contrast this with interactions across groups (i.e. “mixed”). Agents interact more with agents that share their color (majority on majority vs. mixed: t(4)=13.7, *P* < 0.001, minority on minority vs. mixed: t(4)=12.1, *P* < 0.001). Crucially, this is also true for the minority color (Fig. 2*A*, majority on majority vs. minority on minority: t(4)=11.5, *P* < 0.001, *d* = 3.8, post hoc power > 99%). Next, we consider the dual choice evaluation to get clean measure of the group bias of the agents’ actions. As visible in Fig. 2*B*, agents of both colors show a bias to interacting with the agent that shares their respective color. Crucially, this difference decays over time. Fig. 2 *C* and *D* show that this bias depends on how much interactions the two groups have on the mixed server. For groups that never encounter the other group, the group bias is maximal, whereas the group bias gets smaller the more out-group agents are added to the mixed server [overall bias (intercept): F(4,50)=4,262.4,P<0.001, effect of out-group interaction: F(4,50)=600.3,P<0.001].

**Fig. 3.**
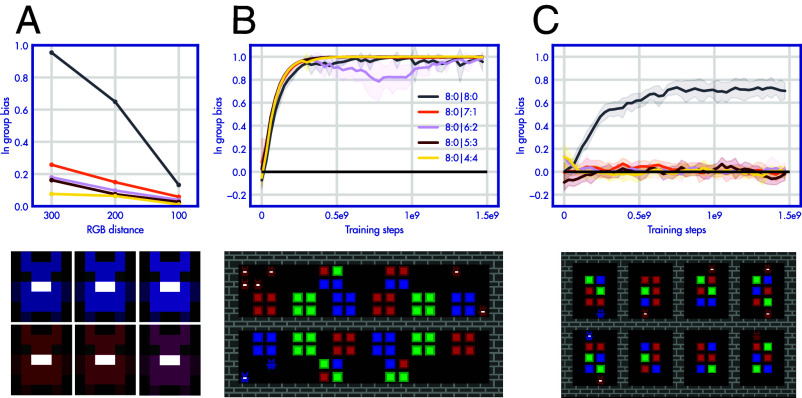
Group bias depends on perception and experience. (*A*) The magnitude of the group bias (as in 1D) for 3 different color differences between the groups. Reducing the RGB distance between groups reduces the group bias. (*B* and *C*) Depicts the same experiment as in Fig. 1 with two different environment layouts. When agent groups are separated by a wall, they cannot interact and the group bias is maximal. When interactions are strongly encouraged by the environment structure (7 out of 8 cells can only contain two agents if they are from different groups) the group bias is nonexistent.

To investigate the impact of perceptual effects, we repeat this experiment with two settings in which the two groups are more visually similar (two shades of purple). The group bias displayed in the behavior probe shrinks as the two groups are more similar [Fig. 3*A*, interaction effect of RGB distance and effect of out-group interaction: F(4,50)=59.96,P<0.001].

Next, we investigate the quality of interactions that are leading to group bias. In Fig. 3 *B* and *C* we run the same experiment depicted in Fig. 1 with two different environment layouts. In B, agents are separated by a wall that does not allow interactions between groups (but agents can see beyond the wall). Now each group shows a very high group bias [overall bias (intercept): F(4,50)=2,457.4,P<0.001]. This shows that just seeing other agents is not sufficient to gain the relevant experience to overcome the group bias. Unlearning the bias requires positive interactions. In C, the environment layout has the opposite effect. The environment is split into 8 cells. In seven of these cells, only agents of two colors can spawn. Only in one cell two agents of the same color can spawn. As now agents mostly interact with agents of the other group (or otherwise no-one at all if they are placed in an empty cell), the group bias is entirely removed [unless for the 8:0 condition in which agents do not even encounter the group on the mixed server, effect of out-group interaction: F(4,50)=109.1,P<0.001]. In combination, these results show that the group bias depends on perceptual factors like visual similarity and the opportunities to interact with the other group.

**Fig. 4.**
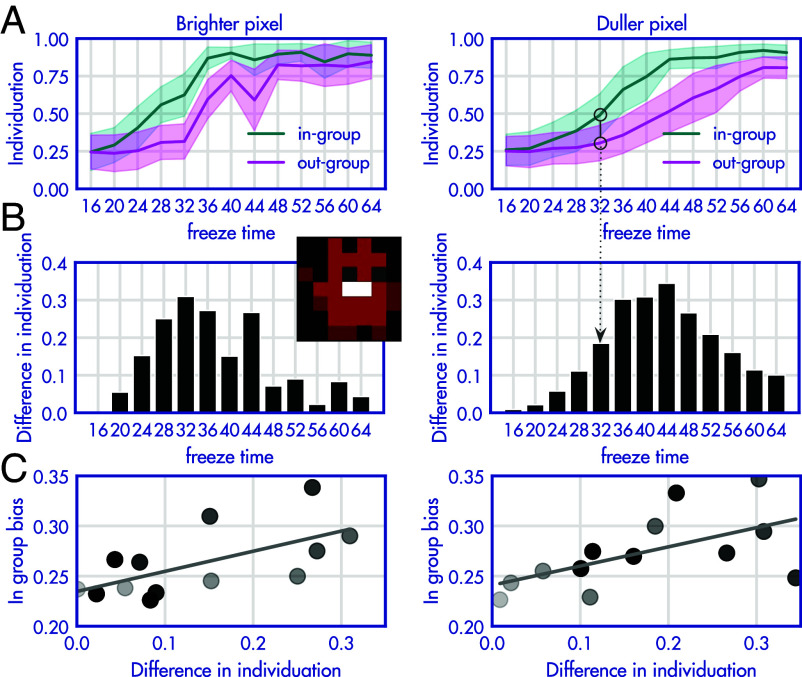
Group bias is exacerbated in situations where individuation is harder. We repeat the experiment (7:1 mixed sampling), but each agent is individuated by a single pixel that makes them uniquely distinguishable (see sprite in center of figure). In each group, interacting with one particular agent leads to a worse interaction experience (because both agents freeze for a period of time we vary across conditions). (*A*) Shows the agents’ ability to discriminate the less rewarding agent against a generic sprite (dual choice evaluation). In the more harmful (high freeze time) conditions agents learn to avoid the less rewarding individual in both their in-group and out-group (high values on the y axis). For low freeze times, agents do not learn to avoid this individual in either group. The gap between the two lines shows the difference in ability to individuate across the in- and out-group (used as y axis in *B*). The *Right* column shows the same data for a setting in which individuation is even harder because the unique pixel is a darker color. Error bars show 95% CIs (across evaluated agents within one training population). (*B*) Summary of the difference in individuation (same data as *A*). (*C*) Correlation between the difference in ability to individuate the harmful individual with the overall magnitude of group bias across runs (markers represent the statistics of one experimental run and are darker for runs with higher freeze time).

Finally, we want to understand how group-bias interacts with agents ability to individuate agents from their own and other group. We repeat the experiment from Fig. 2 in the setting where the mixed server samples 7 from one group and just 1 from the other. In this new setting, each agent is individuated by a single pixel that makes them uniquely distinguishable (see sprites in Fig. 4*B*). Additionally, interacting with one particular agent from each group leads to a worse interaction experience, because both agents freeze for a longer period of time. We vary this time across conditions in order to control how much less rewarding interacting with this one agent is. Fig. 4*A* shows the agents’ ability to discriminate the less rewarding agent against a generic sprite. This was measured via the binary choice probe setup used to measure group bias, but comparing whether the less desirable agent is approached or avoided.

As expected, in conditions where the interaction with the less rewarding individual is more harmful (high freeze), agents learn to avoid (high individuation values) the less rewarding individual. This ability to individuate is tested separately in the in-group and out-group of each respective agent. Generally, we see that accurate individuation is harder in the out-group (lower individuation values for the out-group). At high levels of freeze time, the negative learning signal provided by the less rewarding interactions is so strong, that is it possible to learn who the less desirable agent is. This is true even in the out-group to which one has less exposure. Inversely, when the individual freeze-time is low, agents do not learn to avoid this individual at all (in either group). Fig. 4*A* therefore shows a small gap between the ability to individuate in the in-group and out-group for both the highest and lowest values of freeze time. However, for intermediate amounts of freeze time, there is a difference between the ability to individuate in the in vs. the out-group (due to the different amounts of exposure) (shown in Fig. 4*B*).

We repeat this experiment in a setting in which individuation is further complicated because the unique pixel is a darker color (shown in the *Right* column of Fig. 4). This results in the distribution of values in B being shifted to the right on the x axis. Thus, a duller individuation pixel makes individuation generally harder. As the ability to discriminate is diminished, higher values of freeze time are needed to achieve the same level of discrimination (compared to the brighter pixel).

In combination, we interpret the diminished ability to discriminate to be due to two different types of ‘difficulty’: Individuation is easier if the reward consequences are higher, and individuation is harder if it is perceptually relying on more subtle features.

Interestingly, the difference in ability to individuate the harmful individual across groups is correlated with the overall magnitude of group bias across runs (Fig. 4*C*, *r* = 0.63, *P* = 0.021 and *r* = 0.58, *P* = 0.038, respectively for the easier and harder condition, both *n* = 13). Despite all runs being conducted in the 7:1 setting, there was some variation of how large the group bias was that groups developed. The correlation shows that runs in which the group bias was larger, were also the runs in which the subpopulations showed a large difference in their ability to individuate (i.e. they are able to individuate the less desirable agent in their group, but not in the out-group). Here, bias against an entire group can be amplified by undue generalization of less desirable actors in that group, combined with a lack of sufficient experience to distinguish individuals. In contrast, in the in-group agents are able to individuate less desirable individuals and do not generalize to the rest of the group because the agents had sufficient exposure to all individuals.

## Discussion

In this study, we demonstrate that group bias can emerge without any intrinsic motivational mechanism to support it. Here, agents with no preexisting knowledge about the world learn to predict and maximize their rewards through coordinated interactions with others. We find that differential experience with agents that simply look different from one another is sufficient to generate an in-group bias where agents preferentially approach and interact with agents that look more similar to themselves. Providing evidence that intrinsic motivation is not necessary for the development of in-group bias, the experiment environment used here was carefully designed so that the biased strategy was not more adaptive, and greater points would not be achieved by having an in-group bias. Providing evidence that the amount of direct experience is responsible for these effects, we find that group bias was broadly inversely proportional to the experience agents gain with the other group. In addition to providing strong evidence that minimal differences in experience are sufficient to generate group bias, these studies showed additional features which may increase or decrease group bias: a) when groups were more easily perceptually identified there was more group bias, b) ability to individuate members of the out-group resulted in less group bias, c) extended opportunity for exposure given optimal conditions eventually eliminated group bias.

Together, these results provide a powerful demonstration that in-group bias can emerge without any motivation for bias. As noted earlier, many psychological models of in-group bias propose that in-group behavior and preference emerge through coalitional evolutionary modules or through implicit or explicit self-enhancement mechanisms. The implication is, that people have in-group behavior because we are biologically prepared to do so, or because it helps with another primitive motivational system. Demonstrating in-group behavior with neural net-based agents that do not have these mechanisms provides powerful evidence that simple cognitive mechanisms of bias can emerge without postulating any of these innate systems. Findings such as these further highlight that relatively mundane structural environmental factors such as exposure rates to various individuals may be sufficient to set the stage for intergroup bias. This is especially true in multiagent settings where social rewards are the result of multiple deciders. Indeed, we found that the opportunity to have positive experiences with members of out-groups was not fully under the control of any individual agent as interaction opportunity was also determined by the behavior of the other agents in the environment. Even when we trained an agent to have 50/50 experiences with members of the two groups, that agent subsequently experienced more in-group interaction when placed in an environment with agents that had in-group bias (*SI Appendix*, Fig. S1). This demonstrates the power of structural forces in maintaining bias and determining the formative experiences of agents that not only do not have an intrinsic motivation to be biased but also previously had the opportunity to develop policies to not have bias.

In addition to demonstrating preferential in-group approach behavior, we further demonstrate that these differences in experience are directly related to the degree that agents can individuate others as opposed to treat others as interchangeable members of groups. Simply put, if agents do not have sufficient experience with individuals they do not have the opportunity to build subordinate representations that allow more nuanced behavior. When it was advantageous for agents to learn which agent had a large opportunity cost of interaction (more time spend frozen after interacting with them), their greater number of opportunities with the in-group allowed them to learn not only that coordinating with in-group agents was on average rewarding but also to learn which agent to avoid in order to get a larger return. Consistent with research on intergroup anxiety (56), with less exposure to the out-group, this individuation took longer and therefore the expected reward for approaching in-group members (minus the one exception) was larger than approaching out-group members (because the agent may accidentally approach the agent with high opportunity cost). As such, the out-group members were treated more stereotypically than the in-group members where individualized representations were possible. It is important to note that we observed these effects with a relatively small manipulation of opportunity cost (the slightly longer time delay). To the degree that outcomes can be punishing directly, it is likely that these effects will amplify, especially if survival depends on accurate representations of individuals.

The contact hypothesis, a cornerstone of social psychology, posits that increased intergroup contact can lead to a reduction in prejudice, especially under optimal conditions (57, 58). The nature of the interaction—characterized by collaboration, equal power dynamics, and mutual respect—determines whether contact fosters understanding or further entrenches biases. Given the design of the present study, a significant amount of contact becomes inevitable over time and accordingly bias decreases. Because agents could never permanently exclude or avoid other agents, and because the reward contingencies remained so that in-group and out-group interactions were equally reinforcing, we provided ideal conditions to eliminate the intergroup biases established in the early phases of the experiment. Yet, it is important to note that the biases emerged early and took a substantial amount of time to eventually diminish. When individuals can select who they interact with they have the opportunity to build environments that minimize interactions with members of certain groups that can lead to not only less experience but distorted beliefs and attitudes (36). Conceptually, however, our results suggest a profound duality: The very mechanisms which can sow the seeds of bias also possess the potential to overcome it.

Although these studies demonstrate that innate motivational biases are not necessary for the generation of in-group bias, it is likely that these preparedness factors can also play a role in the development and maintenance of intergroup behavior. Multiagent deep reinforcement learning provides a powerful tool for directly controlling the motivations of the agents to test these hypotheses. For example, past studies have endowed agents with intrinsic motivations like inequity aversion (59), a sense of reputation (48), a desire to punish according to acquired norms (50), a desire to influence others (60) and other-regarding preferences (61). Note that these systems do not just simply alter the payoffs of the environment, but they are integrated in the learning cognitive architecture of each agent. This way relatively small biases can make a large difference over the course of a whole learning population. Just as additional motivational systems can be added and removed to understand their potential to shape social behavior (and ideally compare results with human behavior, e.g., see ref. 48), the cognitive systems that are thought to underpin social behavior can be articulated and modeled directly to test basic tenets of social cognitive theories. As such, the multiagent reinforcement learning framework can provide a testbed for comparing and contrasting different cognitive models in social psychology.

In conclusion, our results demonstrate the opportunities of this methodological approach for scientific study of social cognition. Future work could explore the interplay of innate motivational factors in context of experience-driven learning, as well as the feedback loops that develop in social interactions. By modeling the psychological and structural forces that can lead to more or less bias, large-scale interventions can be tested for proof of principle or theory development on how to ameliorate group bias.

## Materials and Methods

### Multiagent Reinforcement Learning: Model and Notation.

Formally, we consider multiagent reinforcement learning in partially observable general-sum Markov games (62, 63). In each game state, agents take actions based on their own partial observation of the state space and receive an individual reward. Agents learn a behavioral policy through experience while interacting with one another. We formalize this as follows: an *N*-player partially observable Markov game M defined on a finite set of states S. The observation function $O:S\times \left\{1,...,N\right\}\to R^{d}$, specifies each player’s *d*-dimensional view on the state space.

For each state, each player *i* takes an action from their own set of viable actions $A^{i}$.

After all players take their action $\left(a^{1},...,a^{N}\right)\in A^{1}\times ...\times A^{N}$, the state transitions according to the stochastic transition function.

$T:S\times A^{1}\times \;...\;\times A^{N}\to \Delta \left(S\right)$, where Δ(S) denotes the set of discrete probability distributions over S, and every player receives an individual reward defined as,

$r^{i}:S\times A^{1}\times ...\times A^{N}\to R$ for player *i*.

Finally, let $o^{i}={\left\{O\left(s,i\right)\right\}}_{s\in S}$ be the observation space of player *i*.

Each agent learns, independently through its own experience of the environment, a behavior policy $\pi^{i}:O^{i}\to \Delta \left(A^{i}\right)$ (written $\pi (a^{i}|o^{i})$) based on its own observation $o^{i}=O\left(s,i\right)$ and extrinsic reward $r^{i}\left(s,a^{1},\cdots ,a^{N}\right)$. Each agent’s goal is to maximize a long-term *γ*-discounted payoff defined as follows:

[1] $$\begin{array}{c} V_{\vec{\pi }}^{i}\left(s_{0}\right)=E\left[\sum_{t=0}^{\infty }\gamma^{t}r^{i}\left(s_{t},{\vec{a}}_{t}\right)|{\vec{a}}_{t}∼{\vec{\pi }}_{t},s_{t+1}∼T\left(s_{t},{\vec{a}}_{t}\right)\right]\;. \end{array}$$

### Environment.

Agents play a foraging task implemented as a partially observable Markov game in a 2D-world (Fig. 1). Agents gain reward by collecting resources (red, green, or blue) that stochastically populate the environment. Each episode is populated by 8 agents that spawn at random locations. The action space of the agents includes moving forward and turning in all directions, strafing side to side, and firing their “interaction beam.” If agents walk over any resource they collect that resource in their inventory, and they gain a transparent headband that signals they are “ready to interact” (in Fig. 1 on the rightmost panel, the two agents on the left side wear this headband just below their ears). If one agent zaps another with an interaction beam, they get reward proportional to the dot product of their inventories. This leads to high rewards if the two players collected a lot of the same resources. Note that only nearby agents can be zapped with the interaction beam since it has a limited range (and cannot go through walls). Once an agent’s beam connects with another agent in the ready-to-interact state, both agents freeze for 16 frames and then disappear from the game for 50 frames after which they respawn at one of the starting locations. An episode lasts between 1,000 and 5,000 timesteps, determined stochastically.^*^ See https://github.com/google-deepmind/tabula_rasa_agents for an environment configuration.

### Agent Architecture and Training.

Each instance of the training regime contained two populations population of 8 learning agents each, each agent comprising a separate neural network. The environment is a 2-D world filled with sprites of size 8×8 pixels. Agents observe a window of 11×11 sprites (5 to left and right, 9 forward, 1 back), which creates a 88×88 pixels RGB window, centered on the agents’ current location. For each timestep *s*, each agent *i* in the population produced a policy $\pi^{i}$ and an estimate of the value $V_{\vec{\pi }}^{i}\left(s\right)$ computed by a neural network, implemented on a TPU. The neural networks were trained with an actor-critic formulation, receiving importance-weighted policy updates (64, 65) sampled from a queue of trajectories. These trajectories were created by 1,000 simultaneous environments on central processing units that play the game. The agents received truncated sequences of 100 steps of trajectories in batches of 252.

The neural network’s architecture starts with a visual encoder, a 2D-convolutional neural net with two layers. The first layer has 16 channels, with kernel size and stride size 8 to match the sprites) followed by another convolutional layer with 32 channels and 4 by 4 kernel and stride 1. The visual encoder is followed by a 2-layer fully connected multilayer perception (MLP) with 64 rectified linear unit-neurons in each layer and a long short-term memory (256 units). Finally, there is a policy and a value head. The policy head is a MLP with 256 neurons outputting the probability over the 8 basic actions (movement and interact) and the value head is an MLP with 256 neurons outputting a scalar (value of the current state). finally linear policy and value heads. The inventory which is a vector of size 3, is added to the output of the convolutional layers before entering the 2-layer MLP. We used a discount-factor of 0.99, the learning rate was 0.0004, and the weight of entropy regularization of the policy logits was 0.003. We used the RMS-prop optimizer (learning rate = 0.0004, epsilon = 1e − 5, momentum = 0.0, and decay = 0.99). The agent also minimized a contrastive predictive coding loss (66) in the manner of an auxiliary objective (67) and used PopArt (68). This architecture has been widely used on a range of social multiagent tasks (65).

As illustrated in Fig. 1, the two populations get samples to play on two separate “servers.” On the “in-group server,” all 8 agents are sampled (without replacement) from the same population. For example, the 8 red agents player with other red agents only. The “mixed server” samples from both populations, depending on the condition of the run. In the default condition, it samples 6 players from one color and 2 from another. In this condition, on average, an agent will play half of episodes on the in-group server and half of the episodes on the mixed server. On the mixed server, it has a chance to be either in the majority or minority proportional to the ratio of that server (in this example 6:2).

### Calculating Group Bias via Probe Trials.

Alongside training, agents are evaluated in a small environment that is populated with resources and two agents that wear the “ready to interact” headband (“dual choice evaluation”s in Fig. 3). These two agents cannot move or submit any actions and their position is randomized across episodes. The episode ends after 1,000 steps or when the agent that is being evaluated interacts with one of the two other agents.^†^ In the default setting, the probe setting contains one agent of each color. If agents show a group bias, their choice behavior would deviate chance (0.5 on the y axis). If they show a maximal group bias their value on the y axis would be 0 or 1 depending on the group they are more likely to interact with. Agents are evaluated every 5 million timesteps. For analyses, agents’ responses are binned every 30 million steps and averaged to create a response ratio per agent. Due to the distributed nature of the training regime, the amount of probe episodes run per timebin varies but averages around 50 episodes. This response ratio is then averaged over the agents that were evaluated in the population. In the default setting six agents of the population are evaluated this way and averaged over, in the individuation experiment four agents of the population are evaluated this way. The bias of a group per timebins is the average of the averages obtained for each player. The group bias displayed in Figs. 2*B*–*D*, 3, and 4 and *SI Appendix*, Fig. S1 *A* and *C*–*E* are all based on this probe measure of the group bias. This method aims, similar to a psychology experiment, to observe agents’ behavior under controlled settings in which the behavior is not confounded by the behavior of others (49, 69). Agents do not learn from these evaluations.

### Experiments.

#### Variations of server populations.

The results in Figs. 1 and 2 all vary the ratio of players on the mixed server. When set to 8:0, the mixed server acts identically to the in-group server and never gives the two populations the opportunity to interact. At 4:4, the mixed server has the majority and minority groups with equal size. The larger the minority size (up to 4), the more interactions are possible between the two groups.

#### Calculating group bias in training environment.

The results in Fig. 1*A* and *SI Appendix*, Fig. S1*B* are obtained during training. We log the interactions of each player with each other player. We categorize the interactions into “majority on majority” (6 players interacting among themselves), “minority on minority” (the 2 players interacting with each other), or “mixed” (the red and blue players interacting, no matter which group is currently represented by 6 or 2 players in the environment). We first average the interactions in each category and then divide the resulting count by the sum of counts across all 3 categories. This now gives percentages of interactions for each category. For plotting, we further normalize these percentages by what percentages one would expect by chance, given that randomly behaving agents would not create an in-group distribution across the 3 categories (because the two groups are not equally sized). Therefore, the percentage of the mixed group is divided by 12/44, minority on minority by 2/44 and majority on majority by 30/44. Therefore, a value of 1 on the y axis now represents a frequency not deviating from chance expectation. For statistical inference on the training behavior, we utilize the replication runs from the 6:2 setting from *SI Appendix*, Fig. S1*B*. Each comparison is a two-sample *t* test with *n* = 10.

#### Variations of agent colors.

By default, the red and blue groups have the RGB values of [150,0,0] and [0,0,150]. In Fig. 3*A* we adjust the colors to be more similar in two stages, [150,0,50] and [50,0,150], and [150,0,100] and [100,0,150]. The default experiment (varying the ratio on the mixed server from 8:0 to 4:4) in Fig. 1 is reran in these two color settings.

#### Statistical analysis.

One population contains 8 blue and 8 red agents. We extract probe statistics from each 6 agents of each group. For the data displayed in Fig. 2*B* (same data as *C* and *D*), In Fig. 2 *B* and *C* and *SI Appendix*, Fig. S1*A*, we submit the data into a 2 (player color, not of interest) × 5 (how many players are available for out-group interaction) ANOVA with 6 datapoints in each cell. In Fig. 3*A*, the ANOVA has an additional factor of RBG distance with 3 levels. The values in each cell are the group bias ranging from 0 to 0.5. This means that also the intercept is interpretable (i.e. if a group bias is present across all 5 conditions). We also investigate the simple effects of each condition against 0 (i.e. whether bias overall is present) with a *t* test (with 11 degrees of freedom). For *SI Appendix*, Fig. 1*C*, we used R to calculate a multilevel model with agents nested within the 5 replications (70).

#### Variations of environment layout.

In Fig. 3 *B* and *C*, we also rerun the default experience on two different environment layouts. In one layout, a big wall entirely prevents the two populations from interacting (color-specific spawn points are on each side of the wall). The other environment has 8 cells, 7 of them have one red and one blue spawn point (the eighth contains two of both). This leads to many situations in which the only available interaction is with a member of the other population.

#### Individuation.

The individuation experiments in Fig. 4 were always conducted in the 7:1 setting. In this setup, every of the 8 agents has one pixel in their sprite that individuates them from the others. One agent per population is less desirable to interact with, because their freeze rate (the time that both interacting agents are frozen after an interaction) is elevated (it is 16 timesteps for all other agents). Across runs, we vary the freeze time for that one, less desirable agent. To assess the group bias, we run the probe as in the default experiment on a blue and red sprite (without individuating pixel to represent an average sprite). To measure the ability of an agent to individuate the less desirable agent, we run a probe of the less desirable agent against a sprite of the same color without individuating pixel. Fig. 4*A* shows the agents’ ability to discriminate the less desirable agent from the others (1 meaning always picking the more desirable agents to interact with). This metric is computed for the in-group and out-group. The gap between in-group and out-group is displayed in Fig. 4*B*. This analysis results in two measures per game, the group bias of agents, and difference in individuation (i.e. the ability to individuate the less desirable agent more in their in-group). We assess the relationship between these two variables with Pearson correlation.

#### Replications with only one resource color.

In *SI Appendix*, Fig. S1*A*, we rerun the entire default experiment (spanning the mixed server ratios) from Fig. 1 in an environment setting, in which there is only one resource per episode. This means there is no benefit to coordinating on any color, because there is only one color that can be collected. This rules out the possibility that the two populations develop a different convention from the other population. Thus, if any group bias persists it must be due to familiarity, and not getting higher rewards from one group due to better coordination.

#### Full environment evaluation.

In *SI Appendix*, Fig. S1*B*, we run an evaluation (i.e. no learning) of different agents in the full environment (not the probe environment). During the evaluation, the agent groups are split 4:4. We measure the group bias by subtracting the number of expected within and between group interactions. We run this evaluation for 3 sets of agents. The yellow bar depicts the bias of agents trained only on a 4:4 split server (with no in-group server). The purple bar depicts the bias of the population trained in the default 6:2 setting. Finally, the red bar depicts the bias when agents 4 agents from the 4:4 group (2 red, 2 blue) interact with 4 agents from the 6:2 group (2 red, 2 blue).

#### Replications of the 6:2 setting.

In *SI Appendix*, Fig. S1*C*, we rerun the 6:2 setting from Fig. 1 four additional times.

### Replications with different vision architectures.

In *SI Appendix*, Fig. S1 *D* and *E*, we rerun the 6:2 setting with different vision architectures. For these experiments, we replace the convolutional network with a residual network. Residual networks are usually considered to be more powerful than convolutional networks since they outperform them on benchmark vision tasks (71). We ran several versions of this experiment using residual networks of different sizes (see figure legend). All encoders had three ResNet “layers” with number of channels listed in the figure legend ([16,32,32],[8,16,16], or [4,8,8]). Each layer included max pooling (3×3 kernel with spatial stride 2) and contained 2 blocks.

## Supplementary Material

Appendix 01 (PDF)

## Data Availability

Data and analysis code is available at https://github.com/google-deepmind/tabula_rasa_agents.

## Footnotes

^*^Video of example episode: https://youtu.be/LRMFHvT-P48.

^†^Video of example probe episode: https://youtu.be/un3pXJDcwgU.
