# Supplementary material for: Tabula rasa agents display emergent in-group behavior
Source: Proc Natl Acad Sci U S A. 2025 Jun 16;122(25):e2319947121. doi: 10.1073/pnas.2319947121 (PMC12207463; doi:10.1073/pnas.2319947121)
Supplement: Supplementary file 1 — Appendix 01 (PDF) [file pnas.2319947121.sapp.pdf]

Supplemental Materials for Tabula rasa agents display emergent in-group behavior

Raphael Köster[a,1], **Edgar A. Duéñez-Guzmán**[a], **William A. Cunningham**[b,c,d], **and Joel Z. Leibo**[a,1]

[a]Google DeepMind, London EC4A 3TW, United Kingdom; [b]Department of Psychology, University of Toronto, Toronto, ON M5S 3G3, Canada; [c]Vector Institute, Toronto, ON M5G 1M1, Canada; [c]Schwartz Reisman Institute for Technology and Society, University of Toronto, Toronto, ON M5G 1L7, Canada

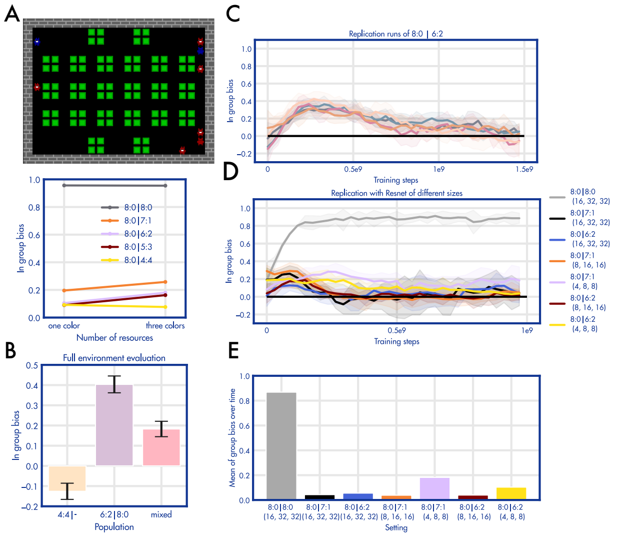

Fig. 1. Supplementary materials: Replications

A. Depicts an environment setting where no coordination is necessary because there is only one resource type per episode. As shown in the lower panel of A, the group bias is smaller but still reproduces the initial pattern (all simple effects $p < 0.001$) when we compare this setting with the default setting. This shows that the effect is not driven by the fact that different groups may coordinate on different colors while they play on the homogenous server. B. Depicts results from an evaluation in the full environment. The population trained on an 4:4 mixed server (and no in-group server) shows a negative bias, whereas the population trained in the 6:2 setting shows a strong in group bias. Critically, mixing these two population results in an overall biased outcome. C. Replicates the 6:2 setting 5 times and shows a similar bias in each run (See main text Fig. 2 for the default experiment), strength of bias across replications $\chi^2 = 149.63$, $p < .001$. D and E. Replicate the bias with different neural network architectures (a ResNet for the vision encoder). While there is always a non-zero bias, the results indicate that there may be a relationship between network size and bias size (reminiscent of (1) who also found larger bias for smaller networks).

1. EA Duéñez-Guzmán, et al., Statistical discrimination in learning agents. *arXiv preprint arXiv:2110.11404* (2021).

[1]To whom correspondence should be addressed. E-mail: rkoster@google.com, jzl@google.com
